# Advances in the Techniques for the Prediction of microRNA Targets

**DOI:** 10.3390/ijms14048179

**Published:** 2013-04-15

**Authors:** Hao Zheng, Rongguo Fu, Jin-Tao Wang, Qinyou Liu, Haibin Chen, Shi-Wen Jiang

**Affiliations:** 1 School of Electrical and Computer Engineering, Georgia Institute of Technology, 225 North Avenue NW, Atlanta, GA 30301, USA; E-Mails: hzheng7@gatech.edu; 2 Department of Biomedical Science, Mercer University School of Medicine, Savannah Campus, 4700 Waters Ave, Savannah, GA 31404, USA; 3 Department of Nephrology, Second Affiliated Hospital, School of Medicine, Xi’an Jiaotong University, Xi’an 710004, Shannxi, China; E-Mail: furongguo@mail.xjtu.edu.cn; 4 Department of Epidemiology, School of Public Health, Shanxi Medical University, 56 Xin Jian Nan Road, Taiyuan 030001, Shanxi, China; E-Mail: wjtxw@yahoo.com.cn; 5 Animal Reproduction Institute of Guangxi University, 100 Daxue Road, Nanning 530005, Guangxi, China; E-Mail: qyliu2002@126.com; 6 Department of Histology and Embryology, Shantou University Medical College, 22 Xinling Road, Shantou 515041, Guangdong, China

**Keywords:** prediction, microRNA, feature selection

## Abstract

MicroRNAs (miRNAs) are small, non-coding, endogenous RNA molecules that play important roles in a variety of normal and diseased biological processes by post-transcriptionally regulating the expression of target genes. They can bind to target messenger RNA (mRNA) transcripts of protein-coding genes and negatively control their translation or cause mRNA degradation. miRNAs have been found to actively regulate a variety of cellular processes, including cell proliferation, death, and metabolism. Therefore, their study is crucial for the better understanding of cellular functions in eukaryotes. To better understand the mechanisms of miRNA: mRNA interaction and their cellular functions, it is important to identify the miRNA targets accurately. In this paper, we provide a brief review for the advances in the animal miRNA target prediction methods and available resources to facilitate further study of miRNAs and their functions.

## 1. Introduction

In addition to DNA methylation and histone modification, epigenetic mechanisms have recently been extended to microRNAs (miRNAs), which are important regulators of gene expression in many biological systems. miRNAs are small, non-coding, endogenous RNA molecules, about 19–24 nucleotides in length that can negatively control their target gene expression post-transcriptionally [1]. This is mainly achieved by recognizing and binding to the 3′ untranslated region of the target messenger RNA (mRNA) sequences [2]. miRNAs have been found to actively regulate a variety of cellular processes, including cell proliferation, death, and metabolism, and therefore, their study is crucial for the better understanding of cellular functions in eukaryotes [3].

Mature miRNAs are incorporated into the RNA-induced silencing complex (RISC), where miRNAs specifically interact with target mRNAs. Approximately one thousand miRNAs have been discovered in humans and are believed to control more than half of the protein coding genes, where a single miRNAs might regulate hundreds of such genes [4]. This one-to-multiple mapping presents a hurdle in accurately identifying the miRNA targets. Furthermore, miRNAs are only partially complementary to their mRNA target sequences. Such imperfections in base matching (e.g., a mismatch or bulge) make it even more difficult to accurately predict the miRNA targets *in silico*[4].

In this paper, we provide a brief review on the advances in the miRNA target prediction methods and available resources. The readers are referred to the literature cited in this review, and the references therein for further details.

## 2. Methods for miRNA Target Recognition

A key step in the identification of miRNA target is the selection of features that are potentially of predictive power. Many researchers are devoted to such an effort, and quite a number of predictive features have been discovered. Such features include dinucleotide composition of flanking sequence [5,6], strong base pairing between the 3′ UTR of mRNAs and the miRNA seed region [7], thermodynamic stability of binding sites [8], evolutionary conservation of binding sites (particularly the seed region) [5,9], secondary structure accessibility [10,11], and host genes expression profiles [12].

The most commonly used predictive features include characteristics in the seed regions and the phylogenetic conservation of miRNA binding sites, and almost all the existing methods take advantage of such features in the algorithm.

For example, by identifying mRNAs with strong base pairing to the 5′ region of the miRNA and evaluating the number and quality of these complementary sites, Lewis *et al*. identified more than 400 regulatory target genes for the conserved vertebrate miRNAs [7]. Likewise, another popular algorithm PicTar [13–17] similarly incorporated seed constraints for the identification of miRNA targets. The new doRiNA database offers computational miRNA target site predictions for human, mouse and worm, and these predictions constitute the most recent update of PicTar predictions [17]. It is notable that some researchers have questioned the universality of the seed assumption, demonstrating that several experimentally confirmed miRNA targets do not seem to meet the seed region criterion. So far, the seed assumption is not unanimously accepted as a method to identify all miRNA targets, and that some relevant miRNA:mRNA interactions might not exhibit the seed region property [18].

With the purpose of enhancing the specificity of prediction for functional target sites, many computational studies also incorporated the evolution conservation [9,14,19–22] or flagged conserved putative targets [8,23]. Particularly, ElMMo [22] incorporated such conservation statistics in a more general, rigorous and miRNA-dependent manner. Also, Friedman *et al*. developed a quantitative method for evaluating evolutionary conservation of binding sites and applied this to the study of vertebrate miRNA targeting With this method, they found three times as many preferentially conserved sites as detected previously, further increasing the known scope and density of conserved miRNA regulatory interactions [9].

Another commonly used feature for target recognition includes the thermodynamic stability of binding sites. It is believed that the formation of a stable miRNA:target binding *in vivo*, to some extent, must be governed by thermodynamic stability. With the rationale that this binding is a process where free energy changes occur through the formation of a miRNA:target duplex, such changes may help detect miRNA targets [24,25]. The computation of energy can vary, but most methods focus only on a particular form of energy (*i.e*., hybridization) [7,14,23,26,27]. For example, Rehmsmeier *et al*. developed a program, named RNA-hybrid, which predicts multiple potential binding sites of miRNAs in large target RNAs based on the thermodynamic stability of binding sites [8].

However, more recently, combining target accessibility and duplex stability [11,28], integrated thermodynamic features for miRNA target prediction demonstrated more effectiveness. In addition, based on the immuno-precipitation (IP) of the RISC components, AIN-1 and AIN-2, Hammell *et al*. presented that total free energy change and target accessibility yielded enrichments in miRISC-enriched transcripts [25,29]. In addition to incorporating accessibility into an energy parameter [28], methods to calculate target accessibility differ, including A/U nucleotides [5,10] and larger nucleotide window to the 5′ of the binding site [29]. More specifically, for example, the Sfold method was used to fold whole 3′ UTR sequences plus 300 nucleotides of adjacent coding sequence for all predicted *C. elegans* transcripts. The output of Sfold was then used to calculate the average accessibility over 25 nucleotide windows flanking each potential microRNA binding site [29].

Expression-based approaches are also becoming popular to elucidate miRNA-mRNA associations. Based on expression profiles of host genes, Radfar *et al*. introduced a new computational method InMiR, which uses a linear-Gaussian model for the prediction of targets of intronic miRNAs [12]. They separated intronic miRNAs into three classes: those that are tightly regulated with their host gene; those that are likely to be expressed from the same promoter but whose host gene is highly regulated by miRNAs; and those likely to have independent promoters. Compared to a method considering only correlation, this method recovered nearly twice as many true positives as the same fixed false positive rate [12]. Engelmann *et al*. recently also showed that entire mRNA expression profiles or large groups of them can be reconstructed only from miRNA expression, and *vice versa*. This introduced a regression model for the prediction of canonical and non-canonical miRNA-mRNA interactions [30].

Furthermore, machine learning algorithms can also be used to intelligently search for the parameters with most predictive power of genuine miRNA binding sites. An example of a method for miRNA target prediction is TargetBoost, which uses machine learning based on a set of validated miRNA targets in lower organisms to create weighted sequence motifs that capture binding characteristics between miRNAs and their targets [31]. Combining genetic programming with boosting, TargetBoost generates a metric that represents the likelihood of a site being targeted by the miRNA.

## 3. Resources for miRNA Target Prediction

Various popular resources for miRNA target predictions are summarized in Table 1. Different miRNA target prediction algorithms can provide differing results, and often researchers need to cross check multiple algorithms to get an additional layer of confidence for the true positive targets. For example, Ryland *et al*. incorporated miRanda [32], microCOSM Targets [33], DIANA-MicroT [27,34] and TargetScan [9] to determine whether the variants detected in mRNA 3′ UTRs occurred within miRNA binding sites [35]. To facilitate that end, starBase was developed to provide a comprehensive exploration of miRNA-target interaction maps from CLIP-Seq and Degradome-Seq data [36]. This allows for a search of commonly agreed upon targets predicted by different algorithms, including TargetScan, PicTar, PITA, miRanda and RNA22 [37]. For example, when TargetScan and PicTar are selected, the database will output target sites predicted by both TargetScan and PicTar programs. This resource greatly facilitates inter-method and inter-database consensus comparison of miRNA targets. In addition, miRTar, an integrated system for miRNA target prediction, enables biologists to easily identify biological functions and regulatory relationships between a group of known/putative miRNAs and protein coding genes. Furthermore, this database delivers perspective information on miRNA targets and their alternatively spliced transcripts [38].

## 4. Next-Generation Sequencing for miRNA Target Identification

With the advances of next-generation sequencing, high-throughput, systematic identification of specific miRNAs targets in a relatively short time became realistic. Several resources using CLIP-seq data to identify miRNA targets were developed, including Piranha [42], CLIPZ [43] and starBase [36]. Piranha [42] provides a utility for peak-calling based on a zero-truncated negative binomial regression model, which is able to incorporate external information to help guide the target identification process. CLIPZ provides a database and analysis environment for experimentally determined binding sites of RNA-binding proteins [43].

## 5. Future Work

Although quite a number of methods and databases have been developed for the identification of miRNA targets, most methods have a false positive rate (FPR) greater than 0.3, which means that the specificity is often lower than 70%. FPR is evaluated as (1-specificity), where specificity is defined as the ratio of the number of true negatives and true negatives plus false positives. Filtering for true positive targets from the large predicted target lists is challenging and time consuming. Although conservation and functional similarities have been taken advantage of to reduce false positives, there is still much room for improvement. Since different miRNA target prediction algorithms still provide varying results, this indicates that such methods also suffer from higher rates of false negatives. As a result, highly accurate prediction algorithms with small false positive and false negative rates need to be further developed. Such algorithms are crucial to studying the exact role of miRNA in signaling pathways, as well as associations with various disease pathways.

To better perform the comparative study of different methods, it is imperative to have some “gold standard” data sets, and quantitatively evaluate different methods based on a fixed set of metrics. The establishment of a gold standard requires strong experimental evidence (reporter assay or western blot analysis) as well as consensus across independent experiments.

## Figures and Tables

**Table 1:** Summary of prediction techniques for miRNA target recognition.

Method	Feature	References	Availability
TargetScan(S)	Database of microRNA targets conserved in 5 vertebrates.	[7,19]	http://genes.mit.edu/tscan/targetscanS2005.html
miRanda	Optimizes sequence complementarity based on position-specific rules and interspecies conservation.	[23,32,39]	http://www.microrna.org
RNA-hybrid	Determines the most favourable hybridization site between two sequences.	[8,40]	http://bibiserv.techfak.uni-bielefeld.de/rnahybrid
PicTar (including doRiNA)	Provides details about 3′ UTR alignments with predicted sites, and links to various public databases.	[13–17]	http://pictar.mdc-berlin.de
TargetBoost	Learns the hidden rules of miRNA-target site hybridization based on machine learning.	[31]	http://www.interagon.com/demo
PITA	Investigates the role of target-site accessibility, as determined by base-pairing interactions within the mRNA.	[11]	http://genie.weizmann.ac.il/pubs/mir07/index.html
ElMMo	Infers miRNA targets using evolutionary conservation and pathway analysis.	[22]	http://www.mirz.unibas.ch/ElMMo2/
Singh’s	Predicts and characterizes 45 miRNAs by genome-wide homology search against all the reported miRNAs.	[41]	http://www.cdfd.org.in/lmg/PDF/imb816.pdf
mirWIP	Employs structural accessibility of target sequences, the total free energy of microRNA:target hybridization, and the topology of base-pairing to the 5 seed region of the microRNA.	[29]	http://ambroslab.org
microCOSM Targets	Web resource containing computationally predicted targets for microRNAs across many species.	[33]	http://www.ebi.ac.uk/enright-srv/microcosm/htdocs/targets/v5/
DIANA-microT 3.0	Individually calculate several parameters for each microRNA and combines conserved and non-conserved microRNA recognition elements into a final prediction score.	[27,34]	http://www.microrna.gr/microT
starBase	Database with intersections among targets by five predictive softwares.	[36]	http://starbase.sysu.edu.cn/clipSeqIntersection.php
InMiR	Uses a linear-Gaussian model, and provides a dataset of 1,935 predicted mRNA targets for 22 intronic miRNAs.	[12]	http://www.plosone.org
miRTar	Identifies the biological functions and regulatory relationships between a group of known/putative miRNAs and protein coding genes.	[38]	http://mirtar.mbc.nctu.edu.tw/human/
